# Faecal microbiota transplantation from young rats attenuates age‐related sarcopenia revealed by multiomics analysis

**DOI:** 10.1002/jcsm.13294

**Published:** 2023-07-13

**Authors:** Xiaoxing Mo, Lihui Shen, Ruijie Cheng, Pei Wang, Lin Wen, Yunhong Sun, Qiang Wang, Juan Chen, Shan Lin, Yuxiao Liao, Wei Yang, Hong Yan, Liegang Liu

**Affiliations:** ^1^ Department of Nutrition and Food Hygiene, Hubei Key Laboratory of Food Nutrition and Safety, MOE Key Lab of Environment and Health, School of Public Health, Tongji Medical College Huazhong University of Science and Technology Wuhan China; ^2^ Department of Health Toxicology, MOE Key Lab of Environment and Health, Tongji Medical College Huazhong University of Science and Technology Wuhan China

**Keywords:** Fecal microbiota transplantation, Gut microbiota, Metabolites, Mitochondrial dysfunction, Muscle, Sarcopenia

## Abstract

**Background:**

Gut microbiota plays a key role in the development of sarcopenia via the ‘gut‐muscle’ axis, and probiotics‐based therapy might be a strategy for sarcopenia. Fecal microbiota transplantation from young donors (yFMT) has attracted much attention because of its probiotic function. However, whether or not yFMT is effective for sarcopenia in old recipients is largely unknown. Thus, we aimed to investigate the effect and mechanism of yFMT on age‐related sarcopenia.

**Methods:**

The fecal microbiota of either young (12 weeks) or old (88 weeks) donor rats was transplanted into aged recipient rats for 8 weeks. Then, muscle mass, muscle strength, muscle function, muscle atrophy, and muscle regeneration capacity were measured. Analysis of fecal 16 s rRNA, serum non‐targeted metabolomic, gut barrier integrity, and muscle transcriptome was conducted to elucidate the interaction between gut microbiota and skeletal muscles.

**Results:**

As evaluated by magnetic resonance imaging examination, grip strength test (*P* < 0.01), rotarod test (*P* < 0.05), and exhaustive running test (*P* < 0.05), we found that yFMT mitigated muscle mass loss, muscle strength weakness, and muscle function impairment in aged rats. yFMT also countered age‐related atrophy and poor regeneration capacity in fast‐ and slow‐switch muscles, which were manifested by the decrease in slow‐switch myofibres (both *P* < 0.01) and muscle interstitial fibrosis (both *P* < 0.05) and the increase in the cross‐section area of myofibres (both *P* < 0.001), fast‐switch myofibres (both *P* < 0.01), and muscle satellite cells (both *P* < 0.001). In addition, yFMT ameliorated age‐related dysbiosis of gut microbiota and metabolites by promoting the production of beneficial bacteria and metabolites—*Akkermansia*, *Lactococcus*, *Lactobacillus*, γ‐glutamyltyrosine, 3R‐hydroxy‐butanoic acid, and methoxyacetic acid and inhibiting the production of deleterious bacteria and metabolites—*Family_XIII_AD3011_group*, *Collinsella*, indoxyl sulfate, indole‐3‐carboxilic acid‐*O*‐sulphate, and trimethylamine N‐oxide. Also, yFMT prevented age‐related destruction of gut barrier integrity by increasing the density of goblet cells (*P* < 0.0001) and the expression levels of mucin‐2 (*P* < 0.0001) and tight junctional proteins (all *P* < 0.05). Meanwhile, yFMT attenuated age‐related impairment of mitochondrial biogenesis and function in fast‐ and slow‐switch muscles. Correlation analysis revealed that yFMT‐induced alterations of gut microbiota and metabolites might be closely related to mitochondria‐related genes and sarcopenia‐related phenotypes.

**Conclusions:**

yFMT could reshape the dysbiosis of gut microbiota and metabolites, maintain gut barrier integrity, and improve muscle mitochondrial dysfunction, eventually alleviating sarcopenia in aged rats. yFMT might be a new therapeutic strategy for age‐related sarcopenia.

## Introduction

With the acceleration of population aging, age and its associated diseases have become a major public health problem in worldwide. Sarcopenia often occurs in the elderly and is characterized by reduced muscle mass, muscle strength weakness, and poor muscle function.^1^ It can cause a series of adverse outcomes, including falls, fractures, and disability, further affecting life quality and even leading to death.^2^ Therefore, exploring effective strategies for sarcopenia is crucial, wherein the regulation of gut microbiota has great potential.

The gut microbiota might be involved in the development of sarcopenia via the ‘gut‐muscle’ axis.^3^ Low muscle mass and strength, poor muscle function, and gut dysbiosis have been simultaneously observed in aged animal models and elderly people.^4^ Gut dysbiosis can disrupt gut barrier function, leading to changes in blood levels of lipopolysaccharide (LPS), indoxyl sulfate, and other metabolites, which ultimately affects muscle metabolism and may play a key role in the development of sarcopenia.^5^ Nevertheless, the types of gut microbiota and metabolites and their mechanistic role in sarcopenia remain unclear.

Interventions targeting the ‘gut‐muscle’ axis might ameliorate sarcopenia‐related phenotypes.^6^ Among these, a probiotic‐based strategy was effective for sarcopenia.^7^ Fecal microbiota transplantation from young donors (yFMT) has also recently attracted extensive attention because of its favourable functional characteristics of probiotics^8^ and has shown protective effects against age‐related behavioural dysfunction.^9^, ^10^ However, the effect and mechanism of yFMT in sarcopenia are poorly understood.

In this study, we transplanted the gut microbiota of either young donors or old donors into aged recipients to explore the effect of yFMT on sarcopenia. Fecal 16 s rRNA analysis and serum nontargeted metabolomic analysis were applied to investigate new gut microbiota and metabolites, and muscle transcriptome analysis was conducted to elucidate the possible mechanism of the gut microbiota and metabolites in muscles.

## Methods

### Animals

Female young rats (*n* = 32, 12 weeks old) were purchased from Vital River Laboratory Animal Center (Beijing, China) and housed in a specific pathogen‐free facility (22 ± 2 °C, 50% ± 5%,12 h dark–light cycle) until 88 weeks of age. Meanwhile, new female young rats (*n* = 8, 12 weeks old) were obtained from the same supplier. Each rat was kept in an individually ventilated cage and provided with sterile standard rodent chow (Cat# SWS9102, Xietong Pharmaceutical Bioengineering Co. Ltd., Nanjing, Jiangsu, China) and sterile drinking water to minimize unwanted exogenous colonization.

### Study design

The aged rats were randomly divided into two following groups: aged rats receiving fecal supernatants from young donor rats (aged yFMT, *n* = 8) and aged rats receiving fecal supernatants from old donor rats (aged oFMT, *n* = 8). In addition, young (young, *n* = 8) and aged donor rats (aged, *n* = 8) were reared to provide fresh fecal pellets. Rats were administered with daily gavage of fecal supernatants (1 mL) for 8 weeks using autoclaved feeding needles to engraft gut microbiota from the donors to the recipients.

At the end of the experiment, fecal pellets were collected to evaluate the colonization efficiency of the donor's gut microbiota in recipients. For the assessment of muscle mass, strength, and function, the rats were then subjected to magnetic resonance imaging (MRI) examination and behavioural tests, including grip strength test, rotarod test, and exhaustive running test. Finally, after fasting for 12 h overnight, the rats were euthanized and their serum and soleus (SOL), gastrocnemius (GC), tibialis anterior (TA), quadricep (QD), extensor digitorum longus (EDL), and plantar (PT) muscles were collected for downstream analysis. The study design is described in Figure S1.

Additional methods were available in supporting information.

## Results

### Fecal microbiota transplantation from young donors rejuvenates skeletal muscle mass, muscle strength, and function in aged rats

**Figure 1:**
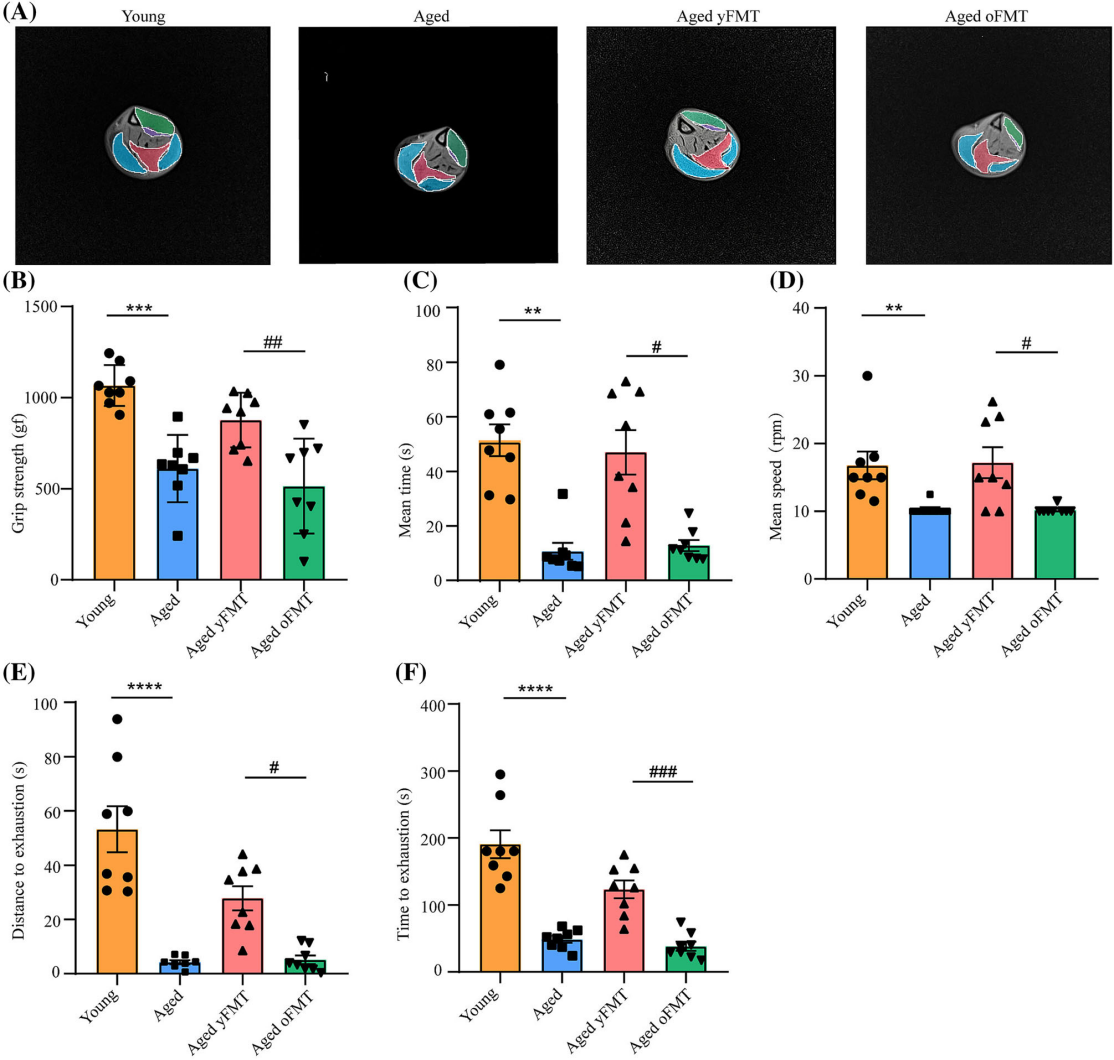
yFMT attenuates age‐related muscle mass loss, muscle strength weakness, and poor muscle function in aged rats. (A) Representative magnetic resonance imaging (MRI) scans of gastrocnemius (GC), tibialis anterior (TA), extensor digitorum longus (EDL), and soleus (SOL) muscles. The areas outlined in red, green, purple, and blue represented GC, TA, EDL, and SOL muscles, respectively. (B) Grip strength (*n* = 8). (C) Mean time in the rotarod test (*n* = 8). (D) Mean speed in the rotarod test (*n* = 8). (E) Distance to exhaustion in the exhaustive running test (*n* = 8). (F) Time to exhaustion in the exhaustive running test (*n* = 8). Data were analysed by *t*‐test (*n* = 8) or Mann–Whitney *U*‐test (*n* = 4). Young versus Aged, *P < 0.05, **P < 0.01, and ***P < 0.001; Aged yFMT versus Aged oFMT, ^#^ *P* < 0.05, ^##^ *P* < 0.01, and ^###^ *P* < 0.001.

No significant differences in weight, food intake, and serum 17β‐estradiol were observed between the aged yFMT rats and aged oFMT rats (Figure S2A–C), indicating that FMT had no effect on the weight, food intake and serum 17β‐estradiol of rats. The muscle mass of QD, TA, EDL, GC, SOL, and PT in the aged rats was lower than that in the young rats (Figure S3A–B). However, the muscle mass of QD, TA, EDL, GC, and SOL in the aged yFMT rats was higher than that in the aged oFMT rats (Figure S3A–B). The protective effect of yFMT treatment on the muscle mass of aged rats was also confirmed by MRI (Figures 1A and S3C, young vs. aged, *P* < 0.01, *P* < 0.0001, *P* < 0.001, and *P* < 0.001; aged yFMT vs. aged oFMT, *P* < 0.05, *P* < 0.001, *P* < 0.001, and *P* < 0.001). Aging also led to poor muscle strength and function, and the reduced muscle strength and function in the aged rats were mitigated by yFMT treatment (Figure 1B–F). The grip strength of the aged yFMT rats was higher than that of the aged oFMT rats (Figure 1B, *P* < 0.01). Meanwhile, in the rotarod test, the time and speed of the aged yFMT rats increased compared with those of the aged oFMT rats (Figure 1C, D, both *P* < 0.05). In addition, in the exhaustive running test, the aged rats receiving yFMT exhibited a significant increase in distance and time compared with those receiving FMT from old donors (oFMT) (Figure 1E,F, *P* < 0.05 and *P* < 0.001). All these findings indicated that yFMT treatment could reverse the decreased muscle mass, muscle strength, and muscle function in aged rats.

### Fecal microbiota transplantation from young donors can ameliorate muscle atrophy and promote muscle regeneration in aged rats

**Figure 2:**
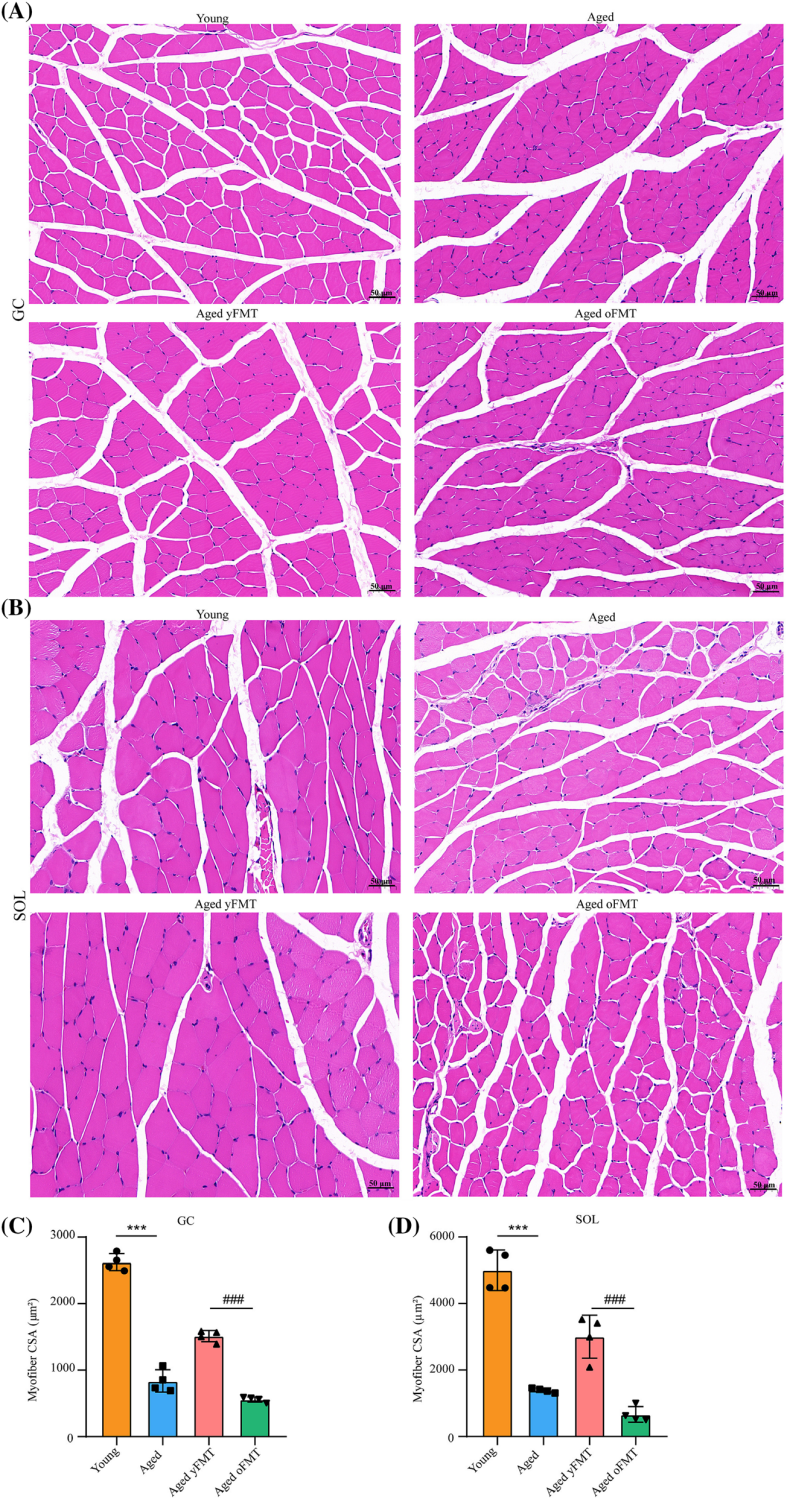
yFMT increases the CSA of myofibres in the muscles of aged rats. (A) H&E staining of GC muscle (scale bar, 50 μm). (B) H&E staining of SOL muscle (scale bar, 50 μm). (C) The cross‐sectional area (CSA) of myofibres in GC muscle (*n* = 4). (D) The CSA of myofibres in SOL muscle (*n* = 4). Data were analysed by Mann–Whitney *U*‐test. Young versus Aged, ***P < 0.001; Aged yFMT versus Aged oFMT, ^###^ *P* < 0.001.

**Figure 3:**
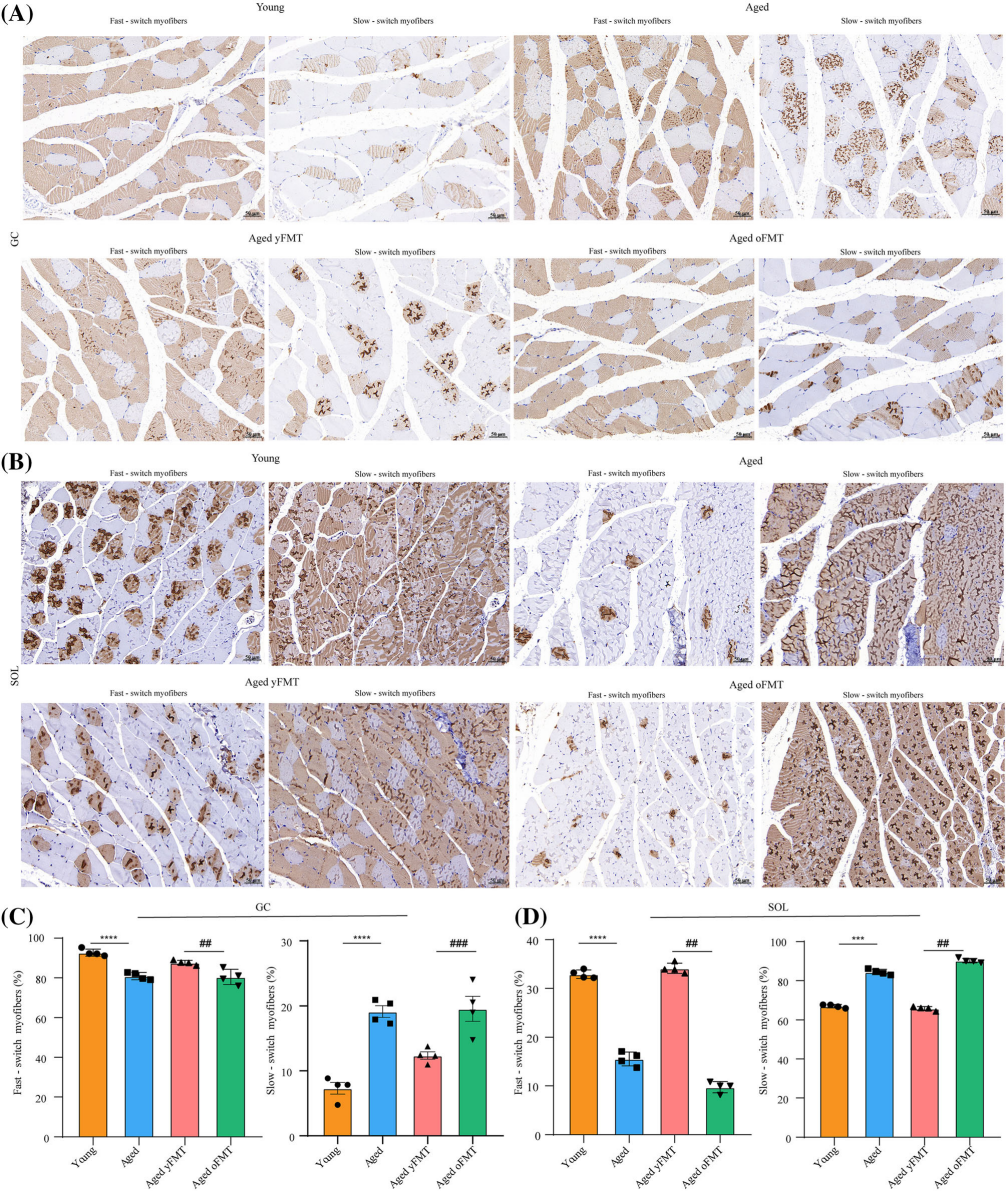
The effects of yFMT on the myofibre types of muscles in aged rats. (A) Immunohistochemical staining of fast‐ and slow‐switch myofibres in GC muscle (scale bar, 50 μm). (B) Immunohistochemical staining of fast‐ and slow‐switch myofibres in SOL muscle (scale bar, 50 μm). (C) Quantification of fast‐ and slow‐switch myofibres in GC muscle (*n* = 4). (D) Quantification of fast‐ and slow‐switch myofibres in SOL muscle (*n* = 4). Data were analysed by Mann–Whitney *U*‐test. Young versus Aged, ***P < 0.001 and****P < 0.0001; Aged yFMT versus Aged oFMT, ^##^ *P* < 0.01 and ^###^ *P* < 0.001.

Based on the results of haematoxylin and eosin (H&E) staining, aging significantly decreased the cross‐sectional area (CSA) of myofibres in GC and SOL muscles (Figure 2A–C, both *P* < 0.001). Contrary to the aged oFMT rats, aged yFMT rats showed an increase in the CSA of myofibres in GC and SOL muscles (Figure 2A–C, both *P* < 0.001). In addition, the fast‐switch myofibres were converted into slow‐switch myofibres in the GC and SOL muscles of aged rats as characterized by the increase in slow‐switch myofibres and the decrease in fast‐switch myofibres (Figure 3A–C, *P* < 0.0001, *P* < 0.001, *P* < 0.001, and *P* < 0.01). These changes could be alleviated by the yFMT. The increase in fast‐switch myofibres and the reduction in slow‐switch myofibres were observed in the GC and SOL muscles of the aged yFMT rats as compared with those of the aged oFMT rats (Figure 3A–C, *P* < 0.001, *P* < 0.01, *P* < 0.01, and *P* < 0.01). Masson staining and Sirius red staining revealed that the interstitial fibrosis of GC and SOL muscles in aged rats was suppressed by the yFMT but aggravated by the oFMT (Figures S4A–D and S5A–D).

In GC and SOL muscles, although the difference was not significant, the number of satellite cells showed a decreasing trend in the aged rats compared with that in the young rats (Figure S6A–D). Meanwhile, the number of satellite cells in the aged yFMT rats was higher than that in the aged oFMT rats as indicated by M‐cadherin (M‐cad) and Pax‐7 immunostaining (Figure S7A–D). In addition, the decreased expression levels of MyoD, myogenin, and IGF‐1 and increased expression levels of atrogin‐1, MuRF, and myostatin in GC and SOL muscles of aged rats were inhibited by the yFMT but promoted by the oFMT (Figure S8A–D). All these results verified the effect of yFMT treatment on inhibiting muscle atrophy and promoting regeneration in aged rats.

### Fecal microbiota transplantation from young donors reinstates gut dysbiosis in aged rats

**Figure 4:**
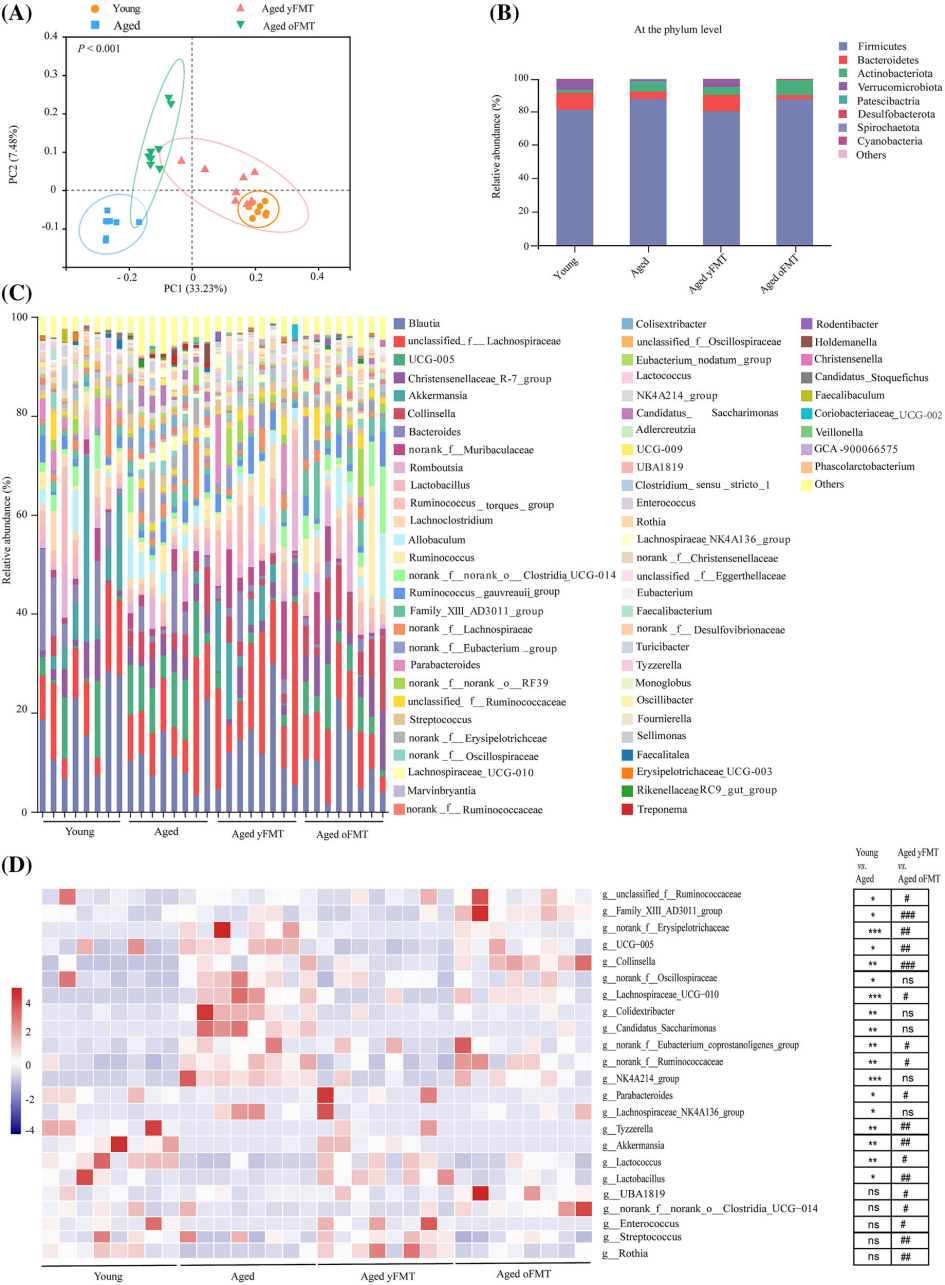
yFMT modulates gut dysbiosis in aged rats. (A) Principal component analysis (PCA) of gut microbiota using unweighted UniFrac method (*n* = 8). (B) Bar plot of gut microbiota at the phylum level (*n* = 8). (C) Bar plot of gut microbiota at the genus level (*n* = 8). (D) Significant changes in gut microbiota at the genus level (*n* = 8). Data were analysed by *t*‐test. Young versus Aged, *P < 0.05, **P < 0.01, ***P < 0.001, and ****P < 0.0001; Aged yFMT versus Aged oFMT, ^#^ *P* < 0.05, ^##^ *P* < 0.01, ^###^ *P* < 0.001, and ^####^ *P* < 0.0001.

At baseline, the gut microbiota of all the aged recipient rats was clustered together and did not show any discernible difference at the phylum level (Figure S9A–B). After the intervention, the α diversity, including Ace index, Chao1 index, and Shannon index of gut microbiota in the aged oFMT rats was higher than that in the aged yFMT rats, which was consistent with the changes of α diversity between aged rats and young rats (Figure S9C). No significant difference was observed in the Simpson index between aged and young rats, or between aged oFMT rats and aged yFMT rats (Figure S9C). In addition, the gut microbiota of the aged yFMT rats significantly differed from that of the aged oFMT rats and was close to that of the young donors (*P* < 0.001, Figure 4A). At the phylum and genus levels, the gut microbiota of the aged yFMT rats exhibited a similar tendency to that of the young donors and distinctly differed from that of the aged oFMT rats (Figure 4B,C). At the phylum level, *Firmicutes* was increased and *Bacteroides* was decreased in the young rats and aged yFMT rats (Figure 4B). However, the opposite variation was discovered in the aged rats and aged oFMT rats (Figure 4B). At the genus level, some beneficial bacteria such as *Parabacteroides*, *Tyzzerella*, *Akkermansia*, *Lactococcus*, and *Lactobacillus* were reduced and other pernicious bacteria including *g_unclassified_f_Ruminococcaceae*, *Family_XIII_AD3011_group*, *norank_f_Erysipelotrichaceae*, *UCG‐005*, *Collinsella*, *Lachnospiraceae_UCG‐010*, *norank_f_Eubacterium_coprostanoligenes_group*, and *g_norank_f_Ruminococcaceae* were enriched in the aged rats relative to those of the young rats. Similar changes were observed in the aged yFMT rats relative to those of the aged oFMT rats (Figure 4C). Nevertheless, the alterations of some bacteria between the young and aged rats, including *norank_f_Oscillospiraceae*, *Colidextribacter*, *Candidatus_Saccharimonas*, *NK4A214_group*, and *g_Lachnospiraceae_NK4A136_group* cannot be transferred by FMT (Figure 4D). These results supported that the gut microbiota of donors is largely transferred to recipients.

### Fecal microbiota transplantation from young donors alters serum metabolites in aged rats

**Figure 5:**
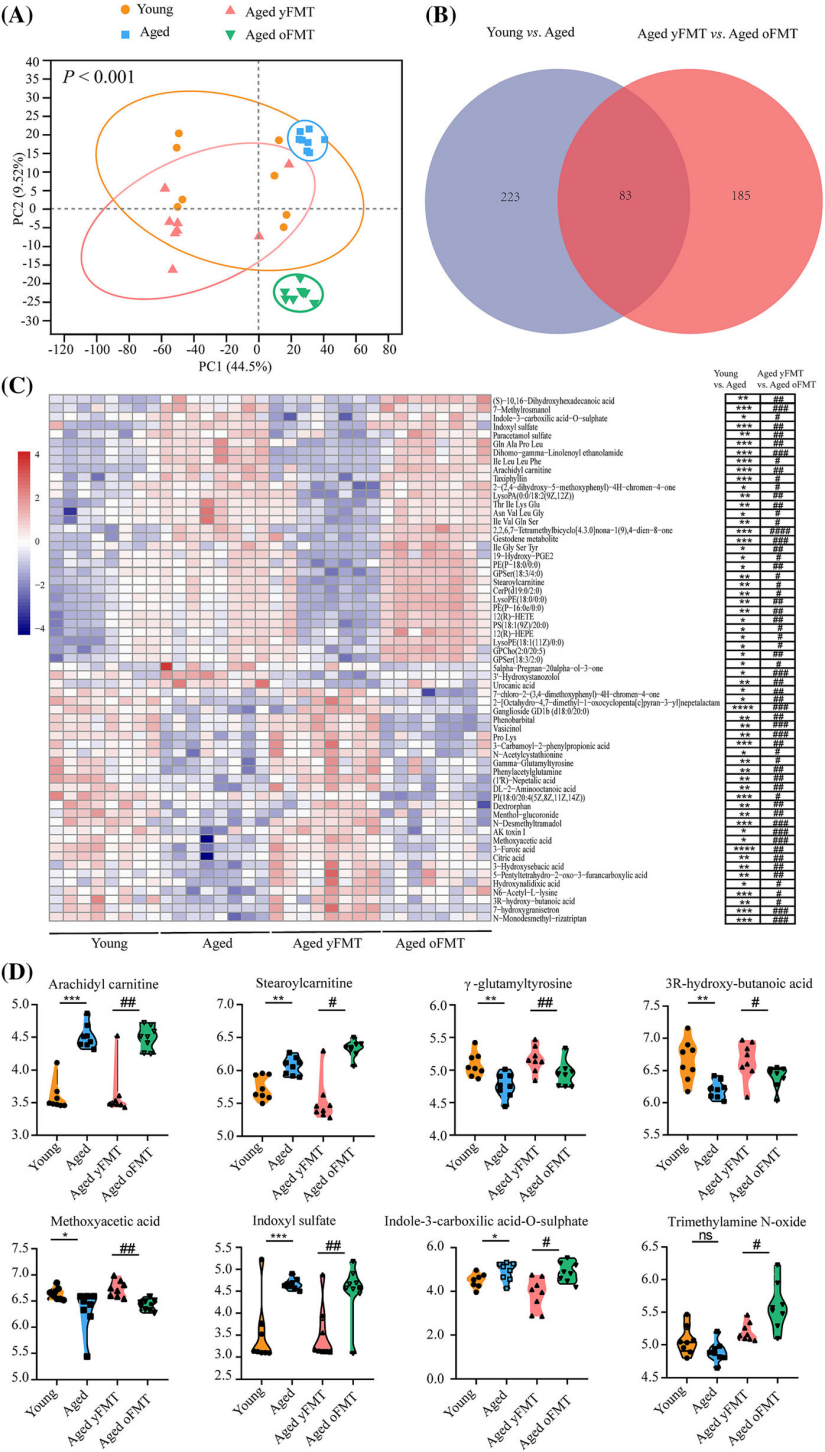
yFMT shapes serum metabolites in aged rats. (A) Partial least squares discriminant analysis (PLS‐DA) analysis of serum metabolite profiles (*n* = 8). (B) Venn diagram showing different serum metabolites among groups (*n* = 8). (C) Heatmap of significantly different serum metabolites that could be restored by FMT treatment (*n* = 8). (D) Violin plots representing different serum metabolites regulated by gut microbiota (*n* = 8). Data were analysed by *t*‐test. Young versus Aged, *P < 0.05, **P < 0.01, and ***P < 0.001; Aged yFMT versus Aged oFMT, ^#^ *P* < 0.05, ^##^ *P* < 0.01, and ^###^ *P* < 0.001. ns, not significant.

Partial least squares discriminant analysis (PLS‐DA) showed significant differences in metabolites between the young and old rats (Figure 5A, *P* < 0.001). Different metabolites were also found between the aged yFMT and oFMT rats, indicating that yFMT treatment could alter metabolites in aged rats (Figure 5A, *P* < 0.001). A total of 1,340 metabolites were obtained, of which 306 were significantly changed between the young and aged rats and 268 were altered between the aged yFMT and oFMT rats (Figure 5B, Tables S2 and S3). Most of these metabolites belong to lipids and organic acids, and 60 of these metabolites were changed in the aged rats but restored to the levels of young donors by yFMT treatment (Figure 5C). Particularly, 17 of these metabolites were regulated by the gut microbiota (Table S4). Among them, lipid‐related metabolites, arachidyl carnitine, and stearoylcarnitine were significantly increased in the aged rats but reduced by yFMT treatment (Figure 5D, young vs. aged, both *P* < 0.01, aged yFMT vs. aged oFMT, *P* < 0.01 and *P* < 0.05). Some organic acids, γ‐glutamyltyrosine, 3R‐hydroxy‐butanoic acid, and methoxyacetic acid were reduced in the aged rats but restored by yFMT treatment (Figure 5D, young vs. aged, *P* < 0.01, *P* < 0.01, and *P* < 0.05; aged yFMT vs. aged oFMT, *P* < 0.01, *P* < 0.05, and *P* < 0.01). Other organic acids, such as indoxyl sulfate and indole‐3‐carboxilic acid‐*O*‐sulphate, were increased in the aged rats (Figure 5D, *P* < 0.001 and *P* < 0.05) but reduced by yFMT treatment (Figure 5D, *P* < 0.01 and *P* < 0.05). In addition, trimethylamine N‐oxide (TMAO) was altered only in the FMT groups, and the level of TMAO was less abundant in the aged yFMT rats than in the aged oFMT rats (Figure 5D, aged yFMT vs. aged oFMT, *P* < 0.05). In summary, these serum metabolites could be modulated by the gut microbiota, demonstrating a close relationship between yFMT treatment and gut microbiota and metabolites.

### Fecal microbiota transplantation from young donors restores impaired gut barrier in aged rats

**Figure 6:**
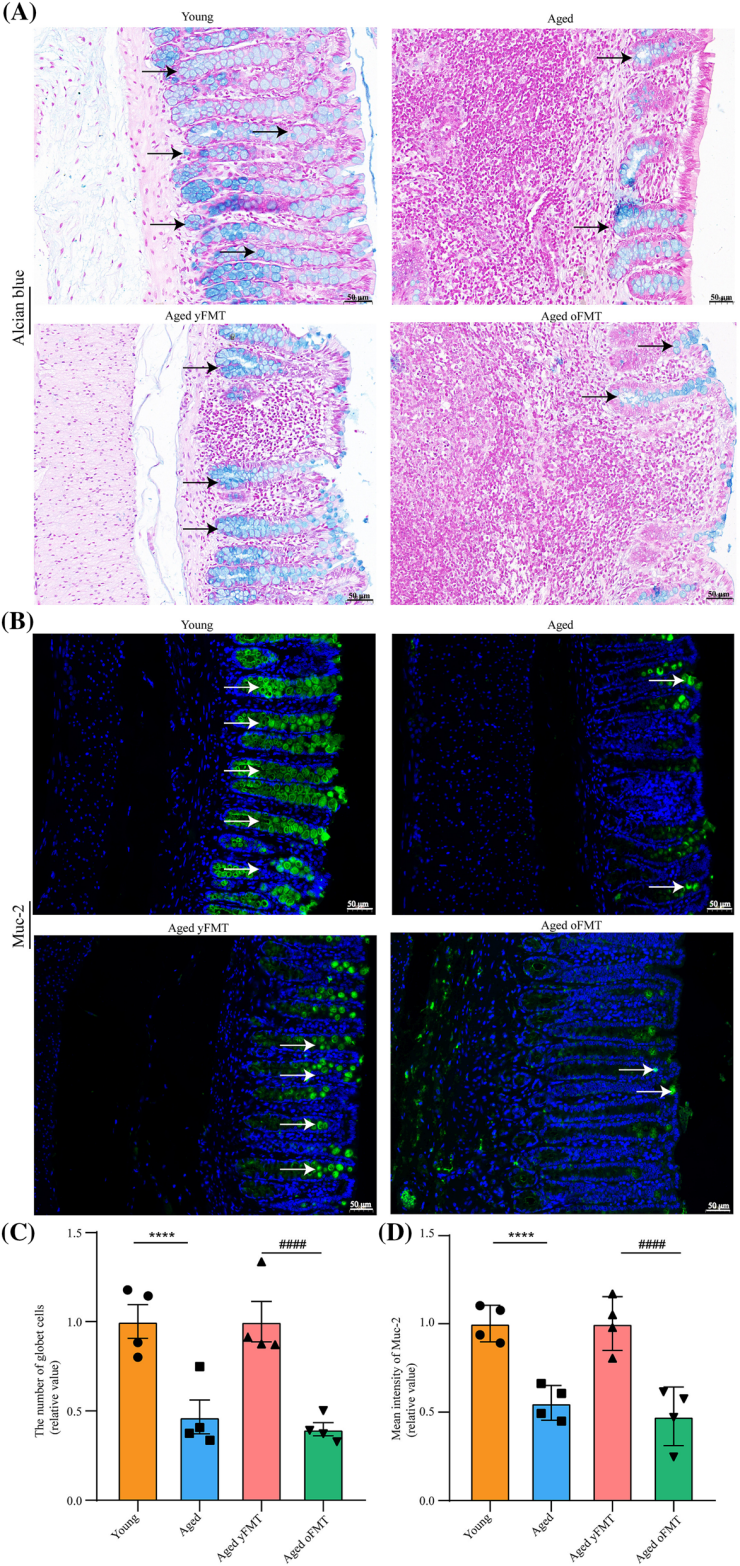
yFMT restores age‐related gut mucosal barrier damage. (A) Alcian blue staining of the colon (scale bar, 50 μm). (B) Representative immunofluorescence images of mucin‐2 (Muc‐2) in the colon (scale bar, 50 μm). (C) The number of goblet cells in the colon (*n* = 4). (D) Quantification of Muc‐2 fluorescence intensity (*n* = 4). Data were analysed by Mann–Whitney *U*‐test. Young versus Aged, ****P < 0.0001; Aged yFMT versus Aged oFMT, ^####^ *P* < 0.0001.

**Figure 7:**
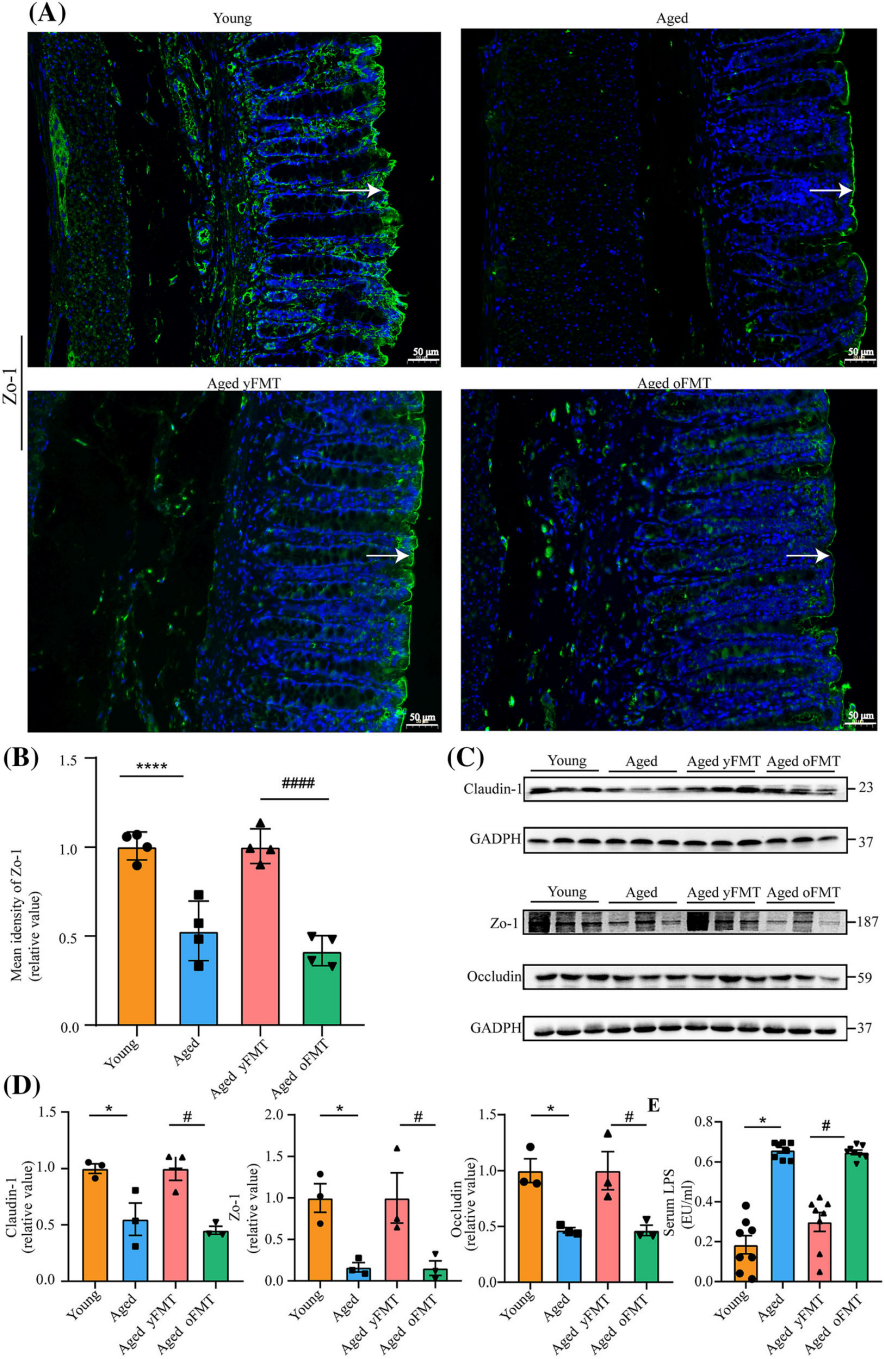
yFMT recovers age‐related gut mechanical barrier impairment. (A) Representative immunofluorescence images of zonula occluden‐1 (Zo‐1) in the colon (scale bar, 50 μm). (B) Quantification of Zo‐1 fluorescence intensity (*n* = 4). (C) Western blot analysis of Zo‐1, occluding, and claudin‐1 in the colon. (D) Quantification of Zo‐1, occluding, and claudin‐1 protein levels (*n* = 3). (E) Serum levels of LPS. Data were analysed by Mann–Whitney *U*‐test. Young versus Aged, *P < 0.05 and ****P < 0.0001; Aged yFMT versus Aged oFMT, ^#^ *P* < 0.05 and ^####^ *P* < 0.0001.

The density of goblet cells and the expression of mucosal protein‐ mucin‐2 (Muc‐2) of the colon were markedly decreased in the aged rats as manifested by Alcian blue staining and immunofluorescence staining, respectively (Figure 6A–D, both *P* < 0.0001). The fluorescence intensity of zonula occludin‐1 (Zo‐1) in the colon was also reduced in the aged rats (Figure 7A,B, *P* < 0.0001). Accordingly, the aged rats had insufficient expression levels of tight junctional proteins Zo‐1, occludin, and claudin‐1 (Figure 7C,D, all *P* < 0.05) and elevated serum LPS levels (Figure 7E, *P* < 0.05). However, these changes were reversed by the yFMT. The aged yFMT rats showed increased goblet cell density and Muc‐2 and Zo‐1 fluorescence intensities in colon compared with the aged oFMT rats (Figures 6A–D and 7A,B, all *P* < 0.0001). Similarly, the increased expression levels of colonic Zo‐1, occludin, and claudin‐1 proteins as well as the decreased serum LPS levels were observed in the aged yFMT rats but not in the aged oFMT rats (Figure 7C–E, all *P* < 0.05). These results suggested that yFMT treatment might maintain gut barrier integrity in aged rats by elevating the density of goblet cells and promoting the expression of Muc‐2 and tight junctional proteins.

### Fecal microbiota transplantation from young donors boosts mitochondrial biogenesis and function in aged muscles

**Figure 8:**
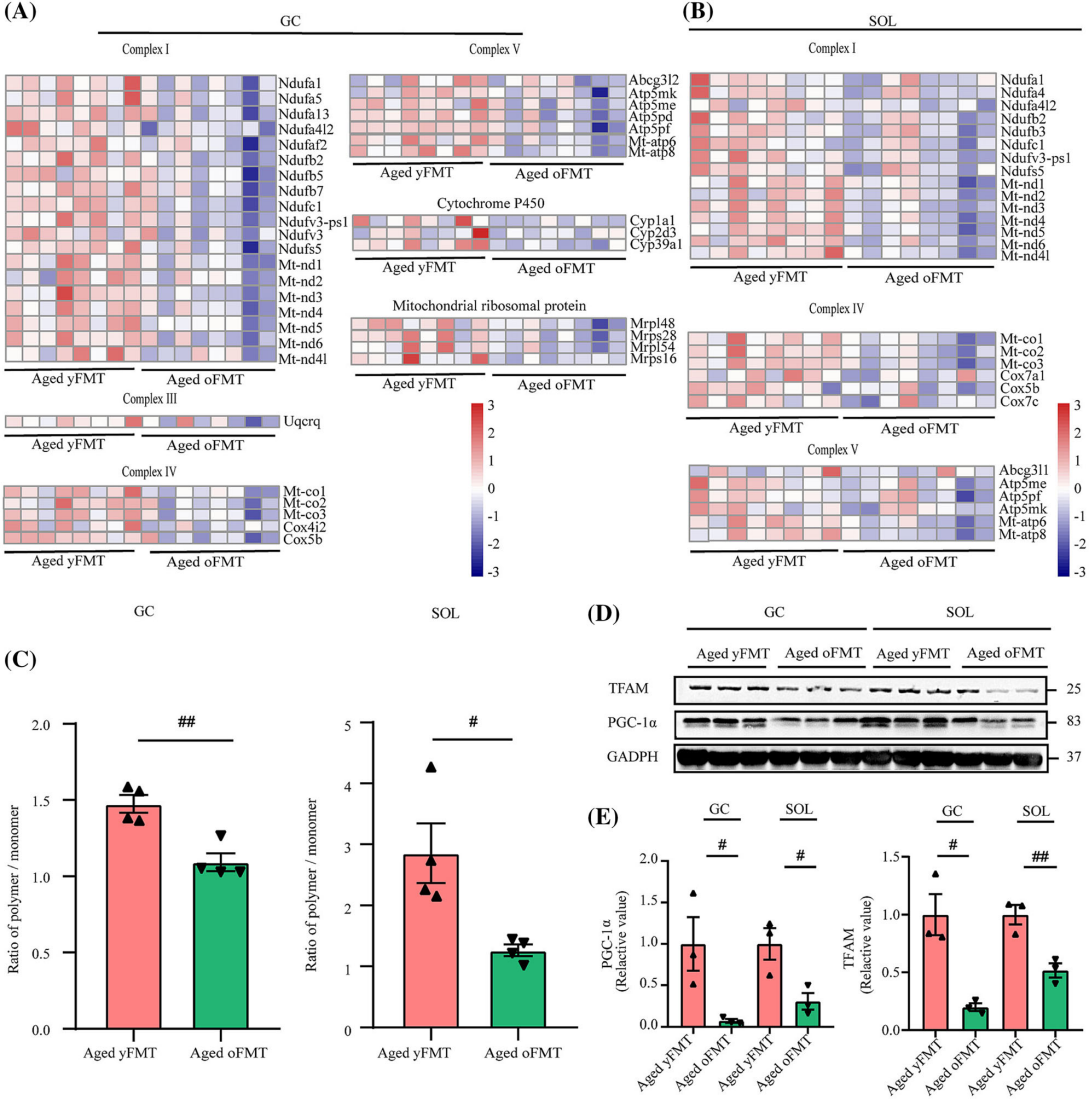
yFMT affects the expression levels of mitochondrial‐related genes in aged muscles. (A) Heatmap of mitochondrial‐related genes in GC muscle (*n* = 8). (B) Heatmap of mitochondrial‐related genes in SOL muscle (*n* = 8). (C) JC‐1 of mitochondria in GC and SOL muscles (*n* = 4). (D) Western blot analysis of peroxisome proliferator‐activated receptor‐γ coactivator (PGC)‐1α and TFAM in GC and SOL muscles. (E) Quantification of PGC‐1α and TFAM protein levels (*n* = 3). Data were analysed by *t*‐test (*n* = 8) or Mann–Whitney *U*‐test (*n* = 4). Aged yFMT versus Aged oFMT, ^#^ *P* < 0.05 and ^##^ *P* < 0.01.

Mitochondria‐related signalling pathways were markedly changed after FMT treatment as indicated by GO analysis (Figure S10). In addition, yFMT treatment elevated the expression levels of respiratory chain‐related genes, including complexes I, II, IV, and V, which were significantly downregulated in the GC and SOL muscles of the aged rats (Figure 8A,B). Moreover, yFMT treatment improved the expression levels of the genes related to complex III, cytochrome P450, and mitochondrial ribosomal protein, which were uniquely decreased in the GC muscle of the aged oFMT rats (Figure 8A,B). JC‐1 staining revealed that yFMT treatment restored the decreased mitochondrial membrane potential of the GC and SOL muscles in the aged rats (Figure 8C, both *P* < 0.01). The expression levels of PGC‐1α and TFAM proteins were significantly higher in the aged yFMT rats than in the aged oFMT rats (Figure 8D, GC: both *P* < 0.05; SOL: *P* < 0.05 and *P* < 0.01). In addition, the mitochondria showed morphological swelling, vacuolar degeneration, and changed cristae structure in the aged oFMT rats. Meanwhile, the mitochondria were compact and round in the aged yMT rats (Figure S11A,B). All these results demonstrated that yFMT treatment could promote the mitochondrial function and biogenesis of GC and SOL muscles in aged rats.

### Crosstalk between gut microbiota, metabolites, mitochondria‐related genes, and sarcopenia‐related phenotypes

Herein, the potential association between gut microbiota, metabolites, mitochondria‐related genes, and sarcopenia‐related phenotypes was investigated using correlation analysis. The yFMT‐induced increase in beneficial bacteria and metabolites such as *Parabacteroides*, *Tyzzerella*, *Akkermansia*, *Lactococcus*, *Lactobacillus*, γ‐glutamyltyrosine, 3R‐hydroxy‐butanoic acid, and methoxyacetic acid was positively associated with muscle mass, strength, and function (Figure S12A). However, the oFMT‐induced increase in deleterious bacteria and metabolites such as *Family_XIII_AD3011_group*, *norank_f_Erysipelotrichaceae*, *UCG‐005*, *Collinsella*, *Lachnospiraceae_UCG‐010*, *g_norank_f_Ruminococcaceae*, arachidyl carnitine, stearoylcarnitine, indoxyl sulfate, indole‐3‐carboxilic acid‐*O*‐sulphate, and TMAO were negatively associated with muscle mass, strength, and function (Figure S12A). Meanwhile, in GC and SOL muscles (Figure S12B,C), the yFMT‐induced increase in beneficial bacteria and metabolites was positively associated with mitochondria‐related genes. On the contrary, the oFMT‐induced increase in deleterious bacteria and metabolites was negatively associated with mitochondria‐related genes. Overall, alterations in gut microbiota and metabolites associated with yFMT might play an important role in the development of sarcopenia.

## Discussion

In this study, we found that yFMT treatment reversed age‐induced muscle mass loss, muscle strength weakness, muscle function impairment, muscle atrophy, and poor muscle regeneration capacity. Results of 16S rRNA and serum metabolomic analyses demonstrated that yFMT treatment reshaped age‐induced dysbiosis of gut microbiota and metabolites. In addition, yFMT treatment attenuated age‐related impaired gut barrier integrity. Transcriptome analysis showed that yFMT treatment mitigated age‐related mitochondrial dysfunction in GC and SOL muscles. Moreover, correlation analysis revealed that yFMT‐induced alterations of gut microbiota and metabolites might be closely related to mitochondria‐related genes and sarcopenia‐related phenotypes.

Muscles are divided into fast‐ and slow‐twitch muscles based on the ratio of fast‐ to slow‐twitch myofibres [S1]. Thus, we selected GC and SOL muscles that are primarily composed of fast‐ and slow‐twitch myofibres, respectively, to investigate the effects of yFMT treatment on different muscle tissues.^11^ Muscle atrophy is manifested by myofibre atrophy and muscle interstitial fibrosis.^12^ In our study, there was a decrease in CSA of myofibres in GC and SOL muscles, suggesting the atrophy of fast‐ and slow‐twitch muscles in aged rats. Herein, the overexpression of Masson staining and Sirius red staining confirmed the interstitial fibrosis of GC and SOL muscles in aged rats. Moreover, muscle atrophy is accompanied by a fast‐to‐slow transition of muscle myofibres.^13^ As shown by the results of immunohistochemical staining, the expression levels of slow‐twitch myofibres were elevated and those of fast‐twitch myofibres were reduced in GC and SOL muscles, which also indicated the atrophy of fast‐ and slow‐twitch muscles in aged rats. Furthermore, age‐related sarcopenia is characterized by reduced regeneration ability. Satellite cells are responsible for adult myofibre regeneration and could not be compensated by other cells [S2]. In line with a previous study [S3], the present work reported the reduced expression levels of Pax‐7 and M‐cad in GC and SOL muscles, indicating the decreased proliferative capacities of satellite cells in aged rats. The decreased expression levels of IGF‐1, MyoD, and myogenin and the increased expression levels of myostatin, atrogin‐1, and MuRF also confirmed the poor proliferative functions of satellite cells in aged rats. IGF‐1 does not only activate the mammalian target of the rapamycin (mTOR) signalling pathway contributing to protein synthesis but also triggers the proliferation and differentiation of satellite cells by promoting the expression levels of MyoD and myogenin [S4]. Contrary to the actions of IGF‐1, myostatin downregulates the expression levels of atrogin −1 and MuRF, which can further inhibit the proliferation and differentiation of satellite cells and reduce muscle growth [S5]. The decrease in slow‐switch myofibres, myostatin, atrogin‐1, MuRF, and muscle interstitial fibrosis and the increase in the CSA of myofibres, fast‐switch myofibres, IGF‐1, MyoD, myogenin, Pax‐7, and M‐cad were found in the aged yFMT rats, revealing that yFMT treatment could alleviate the atrophy of muscle and the impaired proliferative capacities of satellite cells in aged rats.

Consistent with a previous study, we discovered the age‐related increased abundance of *Firmicutes* and reduced abundance of *Bacteroidetes*, which might be related to reduced muscle mass and function.^14^ In addition, aging migh lead to the loss of beneficial bacteria and the accumulation of deleterious bacteria.^15^ In our research, some common beneficial bacteria such as *Parabacteroides*, *Akkermansia*, *Lactococcus*, and *Lactobacillus* were reduced, whereas other deleterious bacteria such as *Family_XIII_AD301_group*, *norank_f_Erysipelotrichaceae*, *UCG‐005*, *Collinsella*, *Lachnospiraceae_UCG‐010*, and *norank_f_Eubacterium_coprostanoligenes_group* were elevated in the aged rats. This deleterious intestinal ecological environment might contribute to the development of sarcopenia; conversely, the beneficial intestinal ecological environment is conducive to the prevention and treatment of sarcopenia.^16^ *Lactobacillus casei* *Shirota* supplementation can alleviate sarcopenia in SAMP8 mice.^17^ *Akkermansia* is positively associated with physical function in patients with sarcopenic cirrhosis.^18^ In addition, *Lactococcus cremoris subsp* can increase muscle mass in middle‐aged mice.^19^ However, as the typical bacteria related to pro‐inflammatory, *Family_XIII_AD3011_group* and *Collinsela* are involved in inflammation‐related disorders, including sarcopenia.^20^, ^21^ In line with these above studies, our correlation analysis also indicated the potential positive effects of *Akkermansia*, *Lactococcus*, and *Lactobacillus* on muscle mass, muscle strength, and muscle function, and the potential negative effect of *Family_XIII_AD3011_group* and *Collinsela* on tmuscle mass, muscle strength, and muscle function. In the present study, we found that the gut microbiota of the aged yFMT rats was significantly different from that of the aged oFMT rats but similar to that of the young donors. Moreover, the aged yFMT rats obtained beneficial bacteria from the young donors, such as *Akkermansia*, *Lactococcus*, and *Lactobacillus*. Meanwhile, the young donors‐related decrease in deleterious bacteria such as *Family_XIII_AD3011_group* and *Collinsella* were also observed in the aged yFMT rats. These changes in gut microbiota might partly explain why the aged rats receiving yFMT showed improvement in sarcopenia.

Gut microbiota‐associated metabolites play a key role in the ‘gut‐muscle axis.’ However, only a few metabolites have been associated with sarcopenia. Consistent with previous studies,^22^ our results showed that indoxyl sulfate and indole‐3‐carboxilic acid‐*O*‐sulphate, metabolites associated with *Family_XIII_AD3011_group* and *Collinsella*, respectively,^21^, ^23^ were increased in the aged oFMT rats. Indoxyl sulfate and indole‐3‐carboxilic acid‐*O*‐sulphate can reduce muscle mass and physical activity.^24^ Furthermore, the increase in serum indoxyl sulfate and indole‐3‐carboxilic acid‐*O*‐sulphate is associated with inflammation and mitochondrial dysfunction in muscles, thereby accelerating muscle atrophy [S6]. Recently, indoxyl sulfate was found to disrupt the intestinal barrier, which is a deleterious factor for sarcopenia [S7]. In this study, we attempted to investigate new microbial metabolites associated with sarcopenia by nontargeted metabolomic analysis. We observed that the level of TMAO was higher in the aged oFMT rats than in the aged yFMT rats. These phenomena might be related to the increase in bacteria associated with TMAO such as *Firmicutes* ^25^ and the decrease in *Akkermansia* and *Lactobacillus*, which could inhibit TMAO production.^26^ Though TMAO, a typical microbial metabolite, is extensively involved in the development of metabolic diseases and aging‐related illnesses, such as obesity, cardiovascular diseases, and Alzheimer's disease [S8, S9], the effect of TMAO on sarcopenia has not been reported. Herein, our correlation analysis showed the potential negative effects of TMAO on muscle mass, muscle strength, muscle function, and mitochondria‐related genes. Emerging evidence has suggested that TMAO could elevate gut permeability and trigger systemic inflammation, which is closely associated with sarcopenia.^27^ TMAO also leads to mitochondrial dysfunction, an important risk factor for muscle atrophy.^28^ Therefore, TMAO may be another biomarker of sarcopenia, but further studies are needed to confirm this hypothesis.

Mucosal barrier and intestinal mechanical barrier are important components to maintain intestinal barrier integrity.^29^ Goblet cells secrete a large amount of mucin, particularly, Muc‐2, the most common mucin, to resist the damage of pathogenic bacteria and metabolites.^30^ The reduced goblet cells and the downregulated Muc‐2 expression in the colon showed that the mucosal barrier was broken in the aged rats. In addition, the decreased expression levels of tight junction proteins, such as Zo‐1, occludin, and claudin‐1, the main components of the intestinal mechanical barrier [S10], indicated that the mechanical barrier was destroyed in aged rats. Age‐induced gut dysbiosis might be involved in the destruction of intestinal barrier integrity. *Akkermansia* and *Lactobacillus* have been widely proven to restore the intestinal barrier by promoting the expression levels of Zo‐1, occludin, claudin‐1, and Muc‐2, which showed decreased abundance in the aged rats.^31^ Conversely, *Collinsella*, which showed increased abundance in the aged rats, can downregulate the expression levels of Zo‐1, occludin, claudin‐1, and Muc‐2 and elevate gut permeability.^32^ The impairment of the mucosal barrier and intestinal mechanical barrier provides a chance for pathogenic bacteria and metabolites to enter other organs, such as muscles, ultimately affecting muscle structure and function [S11]. Accordingly, the serum levels of LPS were found to increase in the aged rats. LPS is a cell wall component of gram‐negative bacteria, such as *norank_f_Erysipelotrichaceae*.^33^ When the gut barrier is destroyed, LPS is transferred to the serum, leading to systemic inflammation and ultimately affecting myogenesis.^34^ LPS can also cause protein metabolic resistance and reduce muscle protein synthesis [S12]. In addition, LPS inhibits myotube growth in differentiated C2C12 myotubes by activating NLRP3 inflammasome‐related inflammation.^35^ The overexpression of tight junction proteins and Muc‐2, the increase in goblet cells, and the decrease in serum LPS levels were observed in the aged yFMT rats compared with those in the aged oFMT rats. These findings supported that yFMT treatment restores the age‐induced intestinal barrier damage, further protecting the muscles from the adverse effect of pernicious bacteria and metabolites.

Increasing evidence has elucidated the critical role of the ‘gut–muscle axis in sarcopenia. Nevertheless, the mechanism of how the gut microbiota affects sarcopenia remains unclear. The reduction in fast‐twitch muscle mass and strength in aged rats could be explained by the 30% decrease in mitochondrial production and complex III electron leakage‐induced ROS generation [S13]. In line with this study, our results revealed that mitochondria‐related genes were significantly downregulated in the GC muscle of aged rats, proving that mitochondrial dysfunction is the crucial mechanism for fast‐twitch muscle atrophy. However, the role of mitochondrial function in slow‐twitch muscle has not been discussed. In this study, mitochondria‐related genes were downregulated in the SOL muscle of aged rats, implying that mitochondrial dysfunction also occurred in slow‐twitch muscles and might be an important mechanism for slow‐twitch muscle atrophy. In addition, we found that the mitochondria‐related genes of GC and SOL muscles were significantly upregulated in the aged yFMT rats than in the aged oFMT rats, indicating that yFMT treatment could attenuate the mitochondrial dysfunction of fast‐ and slow‐twitch muscles in aged rats. PGC‐1α and TFAM are important factors affecting mitochondrial biogenesis [S14]; the promoting effect of yFMT treatment on these factors suggested that it could enhance the production of mitochondria. yFMT treatment also improved complexes I–V, cytochrome P450, mitochondrial membrane potential, and normal mitochondrial morphology, which is a key foundation for mitochondria to exert biological function [S15]. Thus, these findings confirmed that yFMT treatment restores mitochondrial function. *Lactobacillus* supplementation for aged mice ameliorates muscle atrophy by increasing the expression of PGC‐1α and TFAM in muscles.^36^ Conversely, indoxyl sulfate reduces mitochondrial biogenesis and mitochondrial membrane potential in C2C12 cells, thereby possibly accelerating muscle atrophy.^37^ Similarly, TMAO induces mitochondrial dysfunction by affecting complex IV activity.^38^ In line with the above studies, our correlation analysis also showed the potential positive effects of *Lactobacillus* and *Akkermansia* on mitochondrial‐related genes and the potential negative effects of indoxyl sulfate and TMAO on mitochondrial‐related genes. In summary, the yFMT‐induced balance of gut microbiota and metabolites is closely associated with muscle mitochondrial function, further affecting the development of sarcopenia.

Some previous studies have suggested that the receptors treated with antibiotics might promote the colonization of the donor's gut microbiota [S16]. However, recent studies found that pretreatment with antibiotics before FMT cannot increase the overall similarity of the recipient's microbiota to that of the donor's [S17]. In addition, antibiotic treatment can cause glucose intolerance and induce metabolic disorders [S18]. These entangling metabolic side effects may affect muscle function. Then, to make matters worse, aged rats failed to tolerate the antibiotic treatment and died [S19]. Cleaning the intestine with laxatives such as polyethylene glycol (PEG) is an alternative method to antibiotics for depleting the microbiota before FMT [S20]. However, PEG leads to electrolyte disorders, dehydration, and pathological changes in the colonic mucosal layer, which may cause diarrhoea and increase the risk of death in elderly rats [S21]. Furthermore, the gut microbiota derived from recipient rats did not show any discernible difference at baseline. Therefore, we directly transferred the fecal supernatants without antibiotics or PEG pretreatment before FMT.

However, there may be some possible limitations in this study. Though there are large variations in the gender prevalence of sarcopenia, it is known that muscle mass and strength are reduced in postmenopausal women [S22]. In adolescence and middle age, men's muscle mass is higher than that of women, and their muscle build‐up reduces the possibility of sarcopenia during aging [S23]. Contrary to men, there is no accretion of muscle mass in women during adolescence and pre‐menopause, and the loss of muscle mass is widespread in postmenopausal women because of reduced oestrogen [S24]. Therefore, female rats were selected for our study. Nevertheless, further studies are still needed to explore the effect of yFMT on sarcopenia in male rats. In addition, the association between differential gut microbiota/metabolites and sarcopenia was observed in the present study, however, further experiments are required to confirm the causality between them to completely elucidate the mechanism of yFMT on sarcopenia.

## Conclusions

yFMT treatment is a previously unrecognized strategy for age‐related sarcopenia therapy that could restore the age‐related dysbiosis of gut microbiota and metabolites, repair the gut barrier, and improve mitochondrial dysfunction. The balance of the gut microbiota and metabolites is advantageous in improving mitochondrial dysfunction, which is pivotal in sarcopenia, particularly the increase in *Lactobacillus*, *Lactococcus*, and *Akkermansia* and the decrease in *Collinsella*, *Family_XIII_AD3011_group*, indoxyl sulfate, indole‐3‐carboxilic acid‐*O*‐sulphate, and TMAO.

## Funding

This study was supported by the National Natural Science Foundation of China (82230112) and the Angel Nutrition Research Fund (AF2019001‐3). The authors of this manuscript certify that they comply with the ethical guidelines for authorship and publishing in the *Journal of Cachexia, Sarcopenia and Muscle*.^39^

## Conflict of interest

The authors declare that they have no competing interests.

## Supporting information


**Data S1.** Supporting Information

**Data S2.** Supporting Information

**Data S3.** Supporting Information

**Data S4.** Supporting Information
