# Supplementary material for: Faecal microbiota transplantation from young rats attenuates age‐related sarcopenia revealed by multiomics analysis
Source: J Cachexia Sarcopenia Muscle. 2023 Jul 13;14(5):2168–83. doi: 10.1002/jcsm.13294 (PMC10570072; doi:10.1002/jcsm.13294)
Supplement: Supplementary file 1 — Data S1. Supporting Information [file JCSM-14-2168-s001.docx]

**Fecal microbiota transplantation from young donors attenuates age-related sarcopenia revealed by multiomics analysis**

**Journal of Cachexia Sarcopenia and Muscle**

Xiaoxing Mo^1^, Lihui Shen^1^, Ruijie Cheng^1^, Pei Wang^1^, Lin Wen^1^, Yunhong Sun^1^, Qiang Wang^1^, Juan Chen^1^, Shan Lin^1^, Yuxiao Liao^1^, Wei Yang^1^, Hong Yan^2*^ & Liegang Liu^1*^

^1^ Department of Nutrition and Food Hygiene, Hubei Key Laboratory of Food Nutrition and Safety, MOE Key Lab of Environment and Health, School of Public Health, Tongji Medical College, Huazhong University of Science and Technology, 13 Hangkong Road, Wuhan, 430030, China.

^2^ Department of Health Toxicology, MOE Key Lab of Environment and Health, School of Public Health, Tongji Medical College, Huazhong University of Science and Technology, 13 Hangkong-Road, Wuhan 430030, China.

***Correspondence:** Dr. Liegang Liu, Email: lgliu@mails.tjmu.edu.cn; Dr. Hong Yan, Email: yanhong@mails.tjmu.edu.cn.

**Supplementary methods**

**Fecal collection and** **fecal microbiota transplantation (FMT)**

The rats were habituated for at least 2 weeks before fecal collection at baseline. They were placed in sterile empty cages until defecation, and the cages were sterilized with 75% ethanol before the next fecal collection. For fecal 16S rRNA analysis, about 100 mg of feces were immediately collected and stored at −80 °C.

Fresh fecal pellets were daily collected and pooled in our experiment to ensure high fecal bacterial activity for FMT and prevent the change of gut microbiota composition. About 100 mg of fresh fecal pellets were collected and homogenized in 1.5 mL of sterile normal saline with 0.5 g/L L-cysteine and 0.2 g/L sodium sulfide. The homogenates were centrifuged for 2 min at 2000 rpm and 4 °C, and about 1 mL of fecal supernatants were immediately collected for gavage administration. The fecal supernatant was prepared in an anaerobic workstation.

Intragastric administration, magnetic resonance imaging (MRI) examination, behavior tests, fecal samples and tissues collection were conducted from 8 a.m. to 12 a.m. to reduce the impact of the possible different stages of estrous cycle on experimental parameters.

**Serum collection**

**After fasting for 12 h overnight, rats were anesthetized, and blood was collected from the abdominal aorta. The blood was centrifuged at 3000 rpm for 15 min at 4 °C, and then the supernatants were collected. All materials used for serum collection were endotoxin-free, and the whole experimental process was completed with aseptic technique on a super-clean bench.**

**The levels of serum LPS and 17β-estradiol**

The levels of serum LPS and 17β-estradiol were detected using the Toxinsensor TM LAL endotoxin test kit (Cat#L00350, Genscript, USA) and Elisa kit (Cat#EIA-2693, DRG instrument GmBH, Germany) in accordance with the manufacturer’s guidelines, respectively. **All materials or diluents used for serum LPS detection were endotoxin-free, and the whole experimental process was completed with aseptic technique on a super-clean bench.**

**Mitochondrial membrane potential assay**

The mitochondrial membrane potential of GC and SOL muscles were detected using JC-1 kit following the manufacturer's guidelines. The mitochondria were immediately extracted from fresh GC and SOL muscles, incubated with (1x) JC-1 staining working solution, and detected with a fluorescence spectrophotometer (TECAN Spark, Austria). The ratio of polymer fluorescence intensity to monomer fluorescence intensity represented the change of mitochondrial membrane potential.

**Grip strength test**

Grip strength test was measured using a force transducer (Handpi HP-5N, China). The rats were trained to grasp the transducer bar with their four limbs, and their tails were gently pulled back until the rats released the bar [1]. The measurements were repeated three times.

**Rotarod test**

Each rat was placed on the rotating rod whose speed was gradually increased from 0 rpm to 50 rpm in 5 min [2]. The rotational speed and falling time were recorded, and the trails were repeated three times.

**Exhaustive running test**

Each rat received a 2-day training period and a testing day. On the 1st day, the rats were placed in the treadmill at 0 cm/s. On the 2nd day, the speed of treadmill was stabilized at 10 cm/s. On the 3rd day, the running began at 10 cm/s with 0° incline. The speed was then gradually increased by 5 cm/s, and the slop angle was raised by 2°/3 min until it reached 14° [3]. The exhaustive running time and total distance were recorded when the rats could not return back to the track for more than 20 s.

**16S rRNA sequence analysis**

Fecal microbial DNA was extracted using a DNA kit (TIANGEN Biotech Co. Ltd., Beijing, China) and tested for concentration and quality with NanoDrop2000 (Thermo Fisher Scientific Ltd., Waltham, MA, USA) and agarose gel electrophoresis. The qualified DNA was amplified using V3F and V4R primers, the products were sequenced on Illumina MiSeq platform (Illumina, San Diego, CA, USA), and the reads were processed using Quantitative Insights into Microbial Ecology software package (QIIME, Boulder, CO, USA, V.1.9.1). Operational taxonomic units (OTUs) with 97% similarity of reads were prepared for follow-up analyses.

OTU tables for α diversity, β diversity, and differential abundance analyses were constructed using “phyloseq” (version 1.32.0) and DESeq2 (version 1.28.1) R packages. DESeq2 was run with default settings, and p-values were corrected by Benjamini and Hochberg method. All figures were processed using ggplot2 R package.

**Serum** **nontargeted metabolomic analysis**

Serum metabolites were detected by UHPLC-Q Exactive HF-X equipped with an electrospray ionization source operating in either positive or negative ion mode (Thermo Fisher Scientific, USA) referring to previous paper [4]. Briefly, 100 μL serum were homogenized and extracted with methanol-acetonitrile (1:1, v/v) solution. After centrifugation at 13000 g at 4 °C for 15 min, the supernatants were collected for LC-MS/MS analysis. The chromatographic column was HSS T3 column (100 mm × 2.1 mm i.d., 1.8 μm). The mobile phase A and the mobile phase B in the positive ion mode were 0.1% formic acid and acetonitrile, respectively; and the mobile phase A and the mobile phase B in the negative ion mode were 5 mmol/L ammonium acetate aqueous solution with 0.1% ammonia water and acetonitrile, respectively. The flow rate was 0.25 mL/min, and the solvent gradient changed was 0 – 1.5 min, 5% B; 1.5 – 5.5 min, 5% B → 30% B; 5.5 – 10 min, 30% B → 60% B; 10 – 10.5 min, 60% B → 98%B; 10.5 – 14.5 min, 98% B; 14.5 – 15 min, 98% B → 5% B; 15 – 20 min, 5% B. The conditions of mass spectrum condition were as follows: 3.5 kV in positive mode and 2.8 kV in negative mode; capillary temperature, 400 °C; 20 – 40 – 60 V rolling for MS/MS; 40 psi for sheath gas and 10 psi for auxiliary gas. Full MS resolution was 60000, and MS/MS resolution was 7500. The mass range was 70 – 1050 m/z.

Raw data were preprocessed by Progenesis QI software (Waters Corporation, Milford, USA). After a series of data processing, including baseline filtering, peak identification, integration, retention time correction, and peak alignment, a three-dimensional matrix containing sample information, metabolite name and mass spectral response intensity was obtained. The metabolites were searched and identified from Human Metabolome Database, Metlin, and Majorbio databases. The data were filtered for low-quality peaks, filled with missing values, normalized, conversed by logarithm 10, and finally used for downstream analysis. Partial least squares discriminant analysis (PLS-DA) was applied to identify differences in variables. Differential metabolites were screened by ropls (Version1.6.2) R packages with variable importance in the projection >1.0 and adjusted *P* < 0.05. All figures were processed by ggplot2 R package.

**Muscle transcriptome analysis**

The RNA of GC and SOL tissues was extracted using RNA kit (TIANGEN Biotech Co. Ltd., Beijing, China), and further detected for concentration and quality with NanoDrop2000 (Thermo Fisher Scientific Ltd., Waltham, MA, USA) and agarose gel electrophoresis, respectively. RNA library was constructed using the TruSEQ RNA Library Preparation Kit v2 (Illumina) and sequenced on Illumina Novaseq 6000. Sequence alignment and transcript expression were completed by STAR and RSEM. Differential gene expression was analyzed by DESeq2, and p-values were corrected by Benjamini and Hochberg method. Pathway analysis was performed on Blast2go platform. All figures were generated by ggplot2 R package.

Histological analysis

Colon, GC, and SOL tissues were embedded in paraffin and sliced to 7 – 8 μm. The slices were stained with hematoxylin and eosin (H&E) to evaluate the histopathological changes. Alcian blue staining was used to detect the goblet cells of colon. Sirius red staining and Masson staining were performed on GC and SOL slices to assess fibrosis. Images were captured under a microscope (Olympus IX71, Tokyo, Japan). The cross-sectional area (CSA) of GC and SOL muscles, the number of goblet cells, and the area of fibrosis in GC and SOL muscles were measured by Image J software.

Immunohistochemical staining

In brief, 7 – 8 μm paraffin sections of GC and SOL muscles were incubated with anti-fast myosin skeletal heavy chain (Cat # ab51263, 1:1000, Abcam) or anti-slow myosin skeletal heavy chain (Cat # ab11083, 1:1000, Abcam) primary antibodies, followed by biotin-labeled secondary antibodies, and developed with DAB Kit (Zsbio, Beijing, China). Images were captured under a microscope (Olympus IX71, Tokyo, Japan) and quantified using Image J software.

Immunofluorescence staining

In brief, 7 – 8 μm paraffin sections of colon tissues were incubated with zonula occluden-1 (Zo-1; Cat# 40-2200, 1:500, Thermo Scientific) or mucin-2 (Muc-2; Cat# NBP2-66961, 1:100, Novus) primary antibodies, followed by Alexa Fluor 488-labeled secondary antibody (Cat# Z25306, 1:300, Thermo Scientific) and DAPI (Cat# D9542, 1:10000, Sigma).

In brief, 7 – 8 μm paraffin sections of GC and SOL muscles were cocultured with M-cadherin (M-cad; Cat# PA5-106546, 1:100, Novus) and Pax-7 (Cat# PA5-68506, 1:500, Thermo Scientific) primary antibodies, followed by Alexa Fluor 488-labeled (Cat# Z25306, 1:300, Thermo Scientific), Alexa Fluor 546-labeled (Cat# Z25304, 1:300, Thermo Scientific) secondary antibodies, and DAPI (Cat# D9542, 1:10000, Sigma).

All images were acquired under a fluorescence microscope (Olympus IX71, Tokyo, Japan) and quantified by Image J software.

**Transmission electron micrographs (TEM)**

GC and SOL muscles were dissected immediately and fixed in 2.5% glutaraldehyde at 4 °C overnight. On the next day, the tissues were fixed in 1% OsO4, stained with 1% uranylacetate, and embedded in Epon. After ultramicrotomy, the slices were stained with uranyl acetate and lead citrate, and the mitochondria were imaged. The size of the mitochondria was quantified by Image J software.

**MRI**

The rats were anesthetized with 1% – 2% isoflurane (3% – 4% for induction, 0.2 – 0.3 L/min for maintenance) and scanned using a Siemens 3T scanner (Siemens MAGNETOM, Germany) with small animal coil. T1-weighted images were used for examination with the following parameters: TR/TE =1060/15 ms, FOV read = 64 mm, section thickness = 2 mm, and number of slices = 15. The voxel size of reconstructed images was 0.2 × 0.2 × 2.0 mm^3^. The volumes of TA, EDL, GC, and SOL were calculated by Sante DICOM Editor.

**Quantitative real-time PCR (qRT-PCR)**

Total RNA of GC and SOL tissues was extracted by TRIzol reagent (Invitrogen, CA, USA) and reverse transcribed into cDNA using a cDNA synthesis kit (Invitrogen, CA, USA). cDNA was then used as a template for qRT-PCR using SYBR Green® Premix Ex Taq (Invitrogen, CA, USA) kit with GADPH as a reference. The data were analyzed using 2-ΔΔCT method, and the primer sequences are shown in Table S1.

**Western blot**

Colon, GC, and SOL tissues were homogenized with RIPA lysis buffer containing protease inhibitor cocktail. After centrifugation, the supernatant was collected for protein quantification with BCA kits. Proteins were separated by SDS-PAGE gel and transferred to nitrocellulose membranes. After being blocked with 5% milk, the membranes were sequentially incubated with primary antibodies and second antibodies and detected by Box-HR-E-M imaging system (SYNGENE). The primary antibodies were as follows: claudin-1(Cat # ab15098, 1:1000, Abcam), occludin (Cat# ab216327, 1:1000, Abcam), Zo-1 (Cat# 40-2200, 1:1000, Thermo Scientific), peroxisome proliferator-activated receptor-γ coactivator (PGC-1α; Cat# ab106814,1:2500, Abcam), TFAM (Cat# PA5-29571,1:1000, Thermo Scientific), and GADPH (Cat# 3670S, 1:1000, CST). Quantitative analysis was performed with Image J software.

**Statistical analysis**

Data were tested for normality and homogeneity of variance before downstream analysis. **All comparisons were conducted between the young group and aged group, and between the aged yFMT group and aged oFMT group**. Parametric data between two groups were analyzed by T-test, and nonparametric data between two groups were compared by Mann–Whitney U-test. Correlation analysis was conducted by Spearman's correlation analysis, and correlation heatmap was generated by ggplot2 R package. *P* < 0.05 was regarded as significant. Data were shown as mean ± s.e.m.

**References**

1. Padilla CJ, Harrigan ME, Harris H, Schwab JM, Rutkove SB, Rich MM, et al. Profiling age-related muscle weakness and wasting: neuromuscular junction transmission as a driver of age-related physical decline. Geroscience. 2021;43:1265-1281.

2. Pereira SDC, Benoit B, de Aguiar Junior FCA, Chanon S, Vieille-Marchiset A, Pesenti S, et al. Fibroblast growth factor 19 as a countermeasure to muscle and locomotion dysfunctions in experimental cerebral palsy. J Cachexia Sarcopenia Muscle. 2021;12:2122-2133.

3. Palla AR, Ravichandran M, Wang YX, Alexandrova L, Yang AV, Kraft P, et al. Inhibition of prostaglandin-degrading enzyme 15-PGDH rejuvenates aged muscle mass and strength. Science. 2021;371:eabc8059.

4. Wang X, Sun G, Feng T, Zhang J, Huang X, Wang T, et al. Sodium oligomannate therapeutically remodels gut microbiota and suppresses gut bacterial amino acids-shaped neuroinflammation to inhibit Alzheimer's disease progression. Cell Res. 2019;29:787-803.
