# Supplementary material for: Faecal microbiota transplantation from young rats attenuates age‐related sarcopenia revealed by multiomics analysis
Source: J Cachexia Sarcopenia Muscle. 2023 Jul 13;14(5):2168–83. doi: 10.1002/jcsm.13294 (PMC10570072; doi:10.1002/jcsm.13294)
Supplement: Supplementary file 2 — Data S2. Supporting Information [file JCSM-14-2168-s003.docx]

**Fecal microbiota transplantation from young rats attenuates age-related sarcopeni**a [**revealed by multiomics analysis**](https://pubmed.ncbi.nlm.nih.gov/36272371/)

**Journal of Cachexia Sarcopenia and Muscle**

Xiaoxing Mo^1^, Lihui Shen^1^, Ruijie Cheng^1^, Pei Wang^1^, Lin Wen^1^, Yunhong Sun^1^, Qiang Wang^1^, Juan Chen^1^, Shan Lin^1^, Yuxiao Liao^1^, Wei Yang^1^, Hong Yan^2*^ & Liegang Liu^1*^

^1^ Department of Nutrition and Food Hygiene, Hubei Key Laboratory of Food Nutrition and Safety, MOE Key Lab of Environment and Health, School of Public Health, Tongji Medical College, Huazhong University of Science and Technology, 13 Hangkong Road, Wuhan, 430030, China.

^2^ Department of Health Toxicology, MOE Key Lab of Environment and Health, School of Public Health, Tongji Medical College, Huazhong University of Science and Technology, 13 Hangkong-Road, Wuhan 430030, China.

***Correspondence:** Dr. Liegang Liu, Email: [lgliu@mails.tjmu.edu.cn](mailto:lgliu@mails.tjmu.edu.cn); Dr. Hong Yan, Email: [yanhong@mails.tjmu.edu.cn](mailto:yanhong@mails.tjmu.edu.cn).

**Supplemetary references**

S1. Correa-de-Araujo R, Harris-Love MO, Miljkovic I, Fragala MS, Anthony BWManini TM. The Need for Standardized Assessment of Muscle Quality in Skeletal Muscle Function Deficit and Other Aging-Related Muscle Dysfunctions: A Symposium Report. Front Physiol. 2017;8:87.

S2. Roman W, Pinheiro H, Pimentel MR, Segales J, Oliveira LM, Garcia-Dominguez E, et al. Muscle repair after physiological damage relies on nuclear migration for cellular reconstruction. Science. 2021;374:355-359.

S3. Uezumi A, Ikemoto-Uezumi M, Zhou H, Kurosawa T, Yoshimoto Y, Nakatani M, et al. Mesenchymal Bmp3b expression maintains skeletal muscle integrity and decreases in age-related sarcopenia. J Clin Invest. 2021;131:e139617.

S4. Eggelbusch M, Shi A, Broeksma B C, Vazquez-Cruz M, Soares M N, de Wit G M J, et al. The NLRP3 inflammasome contributes to inflammation-induced morphological and metabolic alterations in skeletal muscle. J Cachexia Sarcopenia Muscle. 2022;13:3048-3061.

S5. Kumagai H, Coelho A R, Wan J, Mehta H H, Yen K, Huang A, et al. MOTS-c reduces myostatin and muscle atrophy signaling. Am J Physiol Endocrinol Metab. 2021;320:E680-E90.

S6. Enoki Y, Watanabe H, Arake R, Fujimura R, Ishiodori K, Imafuku T, et al. Potential therapeutic interventions for chronic kidney disease-associated sarcopenia via indoxyl sulfate-induced mitochondrial dysfunction. J Cachexia Sarcopenia Muscle. 2017;8:735-747.

S7. Huang Y, Zhou J, Wang S, Xiong J, Chen Y, Liu Y, et al. Indoxyl sulfate induces intestinal barrier injury through IRF1-DRP1 axis-mediated mitophagy impairment. Theranostics. 2020;10:7384-7400.

S8. Agus A, Clement KSokol H. Gut microbiota-derived metabolites as central regulators in metabolic disorders. Gut. 2021;70:1174-1182.

S9. Li D, Ke Y, Zhan R, Liu C, Zhao M, Zeng A, et al. Trimethylamine-N-oxide promotes brain aging and cognitive impairment in mice. Aging Cell. 2018;17:e12768.

S10. Chelakkot C, Ghim J, Ryu S H. Mechanisms regulating intestinal barrier integrity and its pathological implications. Exp Mol Med. 2018;50:1-9.

S11. Lefevre C, Bindels L B. Role of the Gut Microbiome in Skeletal Muscle Physiology and Pathophysiology. Curr Osteoporos Rep. 2022;20:422-432.

S12. Ni Lochlainn M, Bowyer R C E, Steves C J. Dietary Protein and Muscle in Aging People: The Potential Role of the Gut Microbiome. Nutrients. 2018;10:929.

S13. Wiedmer P, Jung T, Castro J P, Pomatto L C D, Sun P Y, Davies K J A, et al. Sarcopenia - Molecular mechanisms and open questions. Ageing Res Rev. 2021;65:101200.

S14. Miwa S, Kashyap S, Chini Evon Zglinicki T. Mitochondrial dysfunction in cell senescence and aging. J Clin Invest. 2022;132:e158447.

S15. Chocron ES, Munkacsy EPickering AM. Cause or casualty: The role of mitochondrial DNA in aging and age-associated disease. Biochim Biophys Acta Mol Basis Dis. 2019;1865:285-297.

S16. Staley C, Kaiser T, Beura L K, Hamilton M J, Weingarden A R, Bobr A, et al. Stable engraftment of human microbiota into mice with a single oral gavage following antibiotic conditioning. Microbiome. 2017;5:87.

S17. Freitag T L, Hartikainen A, Jouhten H, Sahl C, Meri S, Anttila V J, et al. Minor Effect of Antibiotic Pre-treatment on the Engraftment of Donor Microbiota in Fecal Transplantation in Mice. Front Microbiol. 2019;10:2685.

S18. Ding N, Zhang X, Zhang X D, Jing J, Liu S S, Mu Y P, et al. Impairment of spermatogenesis and sperm motility by the high-fat diet-induced dysbiosis of gut microbes. Gut. 2020;69:1608-1619.

S19. Kim K H, Chung Y, Huh J W, Park D J, Cho Y, Oh Y, et al. Gut microbiota of the young ameliorates physical fitness of the aged in mice. Microbiome. 2022;10:238.

S20. Le Roy T, Debedat J, Marquet F, Da-Cunha C, Ichou F, Guerre-Millo M, et al. Comparative evaluation of microbiota engraftment following fecal microbiota transfer in mice models: age, kinetic and microbial statusm. Front Microbiol. 2018;9:3289.

S21. Bokoliya S C, Dorsett Y, Panier H, Zhou Y. Procedures for fecal microbiota transplantation in murine microbiome studies. Front Cell Infect Microbiol. 2021;11:711055.

S22. Ormsbee M J, Prado C M, Ilich J Z, Purcell S, Siervo M, Folsom A, et al. Osteosarcopenic obesity: the role of bone, muscle, and fat on health. J Cachexia Sarcopenia Muscle. 2014;5:183-192.

S23. Laurent M R, Dedeyne L, Dupont J, Mellaerts B, Dejaeger M, Gielen E. Age-related bone loss and sarcopenia in men. Maturitas. 2019;122:51-56.

S24. Park S, Yuan H, Zhang T, Wu X, Huang S K, Cho S M. Long-term silk peptide intake promotes skeletal muscle mass, reduces inflammation, and modulates gut microbiota in middle-aged female rats. Biomed Pharmacother. 2021;137:111415.
